# Inequity in India: the case of maternal and reproductive health

**DOI:** 10.3402/gha.v6i0.19145

**Published:** 2013-04-03

**Authors:** Linda Sanneving, Nadja Trygg, Deepak Saxena, Dileep Mavalankar, Sarah Thomsen

**Affiliations:** 1 Department of Public Health, Division of Global Health (IHCAR), Karolinska Institutet, Stockholm, Sweden; 2 Indian Institute of Public Health – Gandhinagar, Ahmedabad, India

**Keywords:** maternal and reproductive health, millennium development goal 5, inequity, disadvantaged populations, social determinants of health, India

## Abstract

**Background:**

Millennium Development Goal (MDG) 5 is focused on reducing maternal mortality and achieving universal access to reproductive health care. India has made extensive efforts to achieve MDG 5 and in some regions much progress has been achieved. Progress has been uneven and inequitable however, and many women still lack access to maternal and reproductive health care.

**Objective:**

In this review, a framework developed by the Commission on Social Determinants of Health (CSDH) is used to categorize and explain determinants of inequity in maternal and reproductive health in India.

**Design:**

A review of peer-reviewed, published literature was conducted using the electronic databases PubMed and Popline. The search was performed using a carefully developed list of search terms designed to capture published papers from India on: 1) maternal and reproductive health, and 2) equity, including disadvantaged populations. A matrix was developed to sort the relevant information, which was extracted and categorized based on the CSDH framework. In this way, the main sources of inequity in maternal and reproductive health in India and their inter-relationships were determined.

**Results:**

Five main structural determinants emerged from the analysis as important in understanding equity in India: economic status, gender, education, social status (registered caste or tribe), and age (adolescents). These five determinants were found to be closely interrelated, a feature which was reflected in the literature.

**Conclusion:**

In India, economic status, gender, and social status are all closely interrelated when influencing use of and access to maternal and reproductive health care. Appropriate attention should be given to how these social determinants interplay in generating and sustaining inequity when designing policies and programs to reach equitable progress toward improved maternal and reproductive health.

Millennium Development Goal (MDG) 5 is focused on reducing maternal mortality and achieving universal access to reproductive health care. Under MDG 5, India has committed to reducing maternal mortality to 108 deaths per 100,000 live births by 2015. The latest estimates of maternal mortality rate (MMR) in India, from 2007 to 2009, show a national average of 212 deaths/100,000 live births, a decline of 89 deaths per 100,000 live births since 2001–2003 (1). However, the same estimates also demonstrate that wide geographical disparities persist. The highest MMR can be found in Assam, where it is 390, and the lowest in Kerala, where it is 81 (1).

India has made extensive efforts to reduce maternal mortality and to increase access to reproductive health care and in some regions much progress has been achieved. However, the progress made has been uneven and inequitable, and many women still lack access to maternal and reproductive health care. In India, as in many other settings, social structures prevent women from having access to maternal and reproductive health care. These structures, defined by the WHO as ‘social and structural determinants of health’, vary between different contexts and influence access to and availability of care differently in different societies.

## Social determinants of health

Maternal and reproductive health is a social phenomenon as much as a medical event, where access to and use of maternal and reproductive health care services are influenced by contextual factors. The failure of reaching the targets of MDG 5 is increasingly being analyzed and discussed in terms of equity and, recently, there have been calls for a greater understanding of the patterns of inequity in health within different contexts (2, 3). Culyer has suggested that because of their vulnerability, disadvantaged groups should be identified as a first step toward rectifying inequities in health (4). Further, there is a need to go beyond identifying single determinants of inequity in health, and to illuminate the inter-relationship between social and structural determinants (2).

**Fig. 1:**
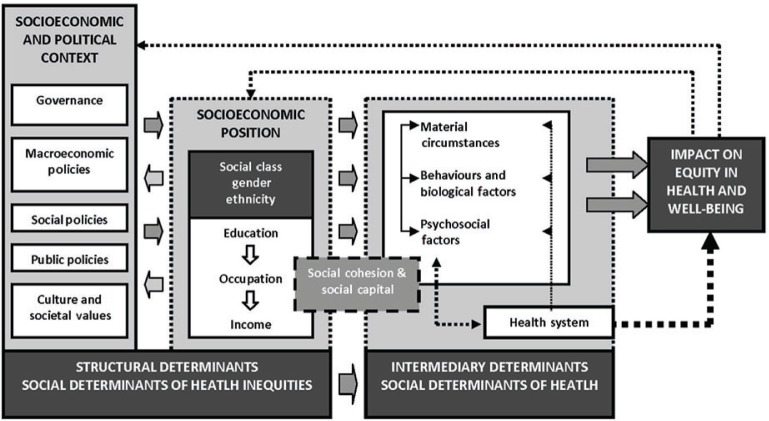
Social determinants of health framework (WHO, 2010).

Inequities in health are not only the unequal distribution of health but also the unfair distribution of health due to unfair or inadequate social arrangements (5). Key features of health inequities are that they are socially produced, systematic in their distribution across the population, and unfair (5). Defining and identifying health inequities thus involves analysis with respect to social justice and the social determinants of health. To enhance the understanding of how inequities in health are rooted in societal structures, the Commission on Social Determinants of Health (CSDH) developed a conceptual framework of the social determinants of health inequities (6). This is an action-oriented framework, applicable to identify entry points for interventions and policy that could reduce inequities in health in a specific setting. It is based on the notion that health inequities emerge from a systematically unequal distribution of power, prestige, and resources among groups in society. The framework is organized into three elements: socioeconomic and political context, structural determinants, and intermediary determinants. Socioeconomic and political context includes governance; macroeconomic, social, and public policy; cultural and societal values; and epidemiological conditions. The second element, structural determinants of health, refers to the interplay between socioeconomic and political context, where structural mechanisms in the society generates a social stratification, which, in the end, results in the socioeconomic position of individuals. The concept of social determinants of health inequities is used to conceptualize the socioeconomic and political context and structural determinants when understood jointly. The structural determinants, or the social determinants of health inequity, operate through a series of intermediary social factors. These intermediary factors include material circumstances such as housing quality and physical environment, psychosocial circumstances such as stressful living conditions and relationships, (lack of) social support and coping styles, and behavioral and biological factors such as lifestyle and genetic factors. The health system is also described as a social determinant of health, particularly since it mediates the differential consequences of ill health (Fig. 1).

There is a vast amount of published papers concerning maternal and reproductive health in India. Some of these papers are focused on issues surrounding equity. However, to our knowledge, there has been no attempt to systematically map the documented sources of inequity and their interrelatedness in maternal and reproductive health in India. The objective of this review was to summarize the evidence on structural and social determinants that generate and sustain health inequity in India to contribute to a more nuanced discussion around achievement of MDG5 in India and how to achieve it equitably. The CSDH framework is used to categorize and explain determinants of inequity with regard to maternal and reproductive health in India.

## Methodology

**Fig. 2:**
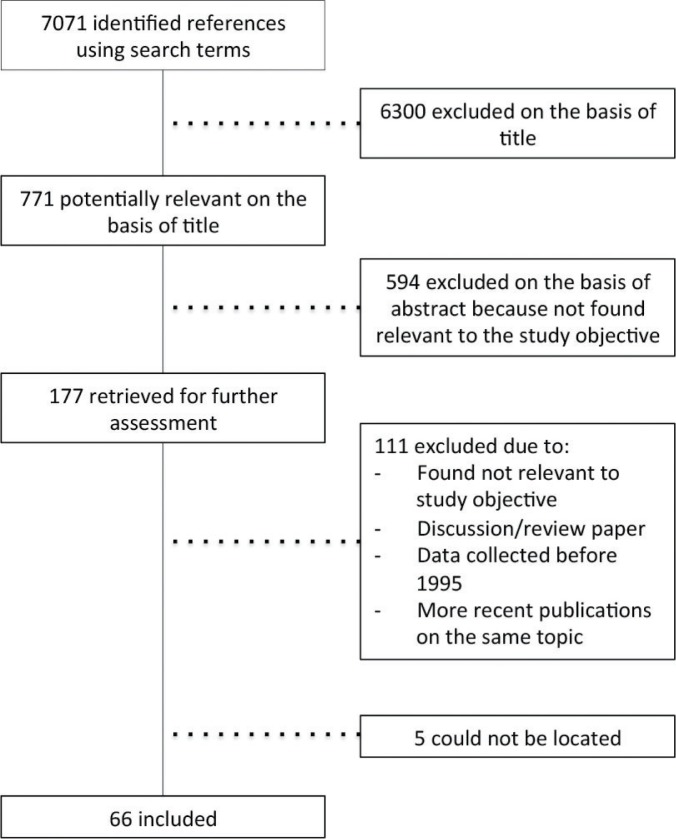
Manual identification of references.

A review of peer-reviewed, published literature was conducted using the electronic databases, PubMed and Popline. The search was conducted by the first author using a carefully developed list of search terms that was designed to capture published papers on: 1) maternal and reproductive health and 2) equity, including possible categories of disadvantaged populations such as place of residence, race/ethnicity, occupation, gender, religion, education, socioeconomic status, and age, amongst others (search terms used can be found in appendix 1). The search terms of the two topics were combined and narrowed down to only include articles on India. The search was delimited to include only those articles published in full in English between January 1st 1995 to October 2012. An overview of the process of identifying literature can be found in Fig. 2. In total, 7,071 articles matched the inclusion criteria. The titles of the 7,071 articles were read manually, and all articles clearly not relevant for the objective of the study were excluded. All articles where relevance could not be determined based on the title were kept. This narrowed down the search to 771 articles. The abstracts of these 771 articles were read manually, and articles not relevant for the objective were excluded. In those articles where it was not possible to determine the relevance of the article based on abstract, the full article was obtained and manually read to determine relevance. This narrowed the sample to 177 articles. All 177 articles were obtained. Then 108 articles were excluded due to:

1. lack of relevance to the study objective;
2. articles being more on the lines of a discussion paper, where data had not been collected;
3. them being review articles;
4. data being collected before 1995; and
5. similar findings on the same topic in a more recent publication.

Many of the articles used data drawn from the three rounds of National Family Health Surveys (NFHS), and when similar findings on the same topic were found in two publications using data from different rounds of the NFHS, only the most recent publication was included. Five articles could not be identified and obtained. Sixty-six articles were included in the study. A thematic content analysis of these articles was conducted using a matrix developed to sort the information obtained from each article (a summary of the matrix can be found in appendix 2). The matrix organized the information provided by each article into these groups: inequity relevance; structural determinant(s) addressed; area of research; study population; setting; method; outcome; intermediary determinant; and explanation/discussion.

## Results

Five main structural determinants emerged from the search as important in understanding equity in the context of India: economic status, gender, education, social status (registered caste or tribe), and age (adolescents). These five determinants are closely interlinked, which is reflected in the literature. In a majority of the articles, economic status is the primary focus; where it is not the main focus, it is often brought up in the discussion. Social status, which in the context of India can be measured with the proxy of ‘caste’, is often discussed in terms of economic status. There is also an overlap between how gender and adolescence generates and sustains inequity and between economic status and education. Income, occupation, ethnicity, and religion are all determinants of equity that are reported in a few of the retrieved articles but no articles were found where these determinants were the primary focus or were discussed in-depth.

As context is essential in understanding the underlying determinants of health inequity, each of these determinants will be preceded by a short description of the current situation in India, including at the subnational level where possible.

### Economic status and health financing

According to the Tendulkar Committee report, which the Indian Government accepted in 2011, the proportion of people living in poverty was estimated at 37% of the population (7). However, poverty levels differ widely across India: in Delhi, Goa, and Punjab they are under 10%, while in some states, such as Bihar and Orissa, they are over 40%. Government health spending is around 1% of GDP, while the total spending on health in India is around 5% of the GDP. India has one of the highest levels of out-of-pocket payments for health care in the world, which imposes a large financial burden on individuals and households (8). This has been argued to be one of the reasons for the inequities in health observed across the country (9). India has an integrated policy framework for human development and public policy as well a great variety of social protection schemes, which reflects an intention to assist the disadvantaged segments of the population (10). Since 2005, the National Rural Health Mission (NRHM) has been running as a national overarching project to approach inequities in health specifically by focusing on rural areas.

Utilization of antenatal care (ANC) and skilled attendance at birth has increased among the general population in India in the last 15 years. However, progress among women belonging to economically disadvantaged segments of the populations has been slow. Results from a study looking at progress based on data from the three rounds of NFHS in 1992–1993, 1998–1999, and 2005–2006 showed that use of ANC services in the whole of India increased by 12 percentage points between 1992 and 2006 but that the increase among the poor was only 0.1 percentage points (11). The same study also showed that the use of skilled birth attendants had increased by 13 percentage points but that only 2 percentage points could be attributed to women belonging to the poorest quintile. This study also showed that there are large differences in progress between states but that the progress among the poor is substantially less than among the non-poor in all states and that the use of skilled birth attendance among the poor remained low across an urban–rural spectrum. A similar large nationwide study, also looking at the differences in use of reproductive health care between the poor and the non-poor, showed that increased utilization has occurred mainly in the non-poor populations (12). Concordance with desired waiting time to the first birth has been shown to be associated with economic status. A study based on data from all over India showed that couples belonging to the richer quintile had twice the odds as couples from the poorest quintile to have concordant desired waiting time to first birth (13). The findings from this study are interpreted as an unmet need for contraception among couples with low economic status (13).

#### Regional and rural/urban differences in health based on economic status

A household survey from Chandigarh Union Territory comparing coverage of maternal health care showed that among the women studied, only 32% of the women living in urban-slum areas had an institutional delivery, compared to 93% of the non-slum urban women, and 79% of the women living in rural areas (14). The average maternal expenditure varies between geographical areas and between providers. However, a study using the National Sample Survey from 2004 showed that a vast majority of the poorest households in the country paid more than 40% of their capacity to pay for maternal health services (15). A community survey from South Delhi showed that direct maternity expenses are high, sometimes exceeding 10% of the annual family income for the poorest (16).

Quality of care in maternal health services has also been shown to differ according to economic and residence status. A cross-sectional study conducted in Andhra Pradesh, Karnataka, Kerala, and Tamil Nadu showed significant differences in the quality of ANC between poor and non-poor groups (17). A study conducted in New Delhi showed that health care providers were unable to meet the national standards on minimal care during pregnancy and delivery in the poorer areas of the city, whereas this did not to seem to be a problem in the higher-income areas of the city (18). A qualitative study that included both rural and urban areas of Maharashtra showed that financial constraints are important when understanding the user's perspective of barriers to maternal health care but that these are also closely linked to perceptions of health care (19). One of the findings from the study was that ANC and institutional delivery are both classified as preventative measures rather than curative and, due to financial constraints, are not prioritized.

The urban population in India is one of the largest in the world, with many living in urban slums. A study on women's reproductive health showed that a significant lower proportion of women living in slum areas compared with women living in non-slum areas had ever used contraceptives, were less likely to use skilled attendants at delivery, and less likely to receive postpartum check-ups (20). A hospital-based study conducted in New Delhi showed that the use of contraceptives among urban poor was 52%, which is similar to contraceptive use in rural areas but below the use in well-educated urban populations (21). Data from the first and third round of the NFHS also showed that progress toward increased use of ANC and institutional delivery is occurring mainly among the urban non-poor, and that progress among urban-slum residents is slow (22). The burden of costs for maternal health care among the population living in slums is often significant. A study on expenditure on maternal health care showed that the poor living in slums in the city of Mumbai spent catastrophically on care, which is assumed to occur when the health expenditure exceeds a proportion (usually 40%) of the total household income. The study also showed that a high proportion of the total spending was spent on informal costs (23). The same study also showed that poor households in the slums were likely to use wage income, as compared to higher income groups that used savings, and borrowed money to pay for maternal health care, which is assumed to increase the risk of both transient and chronic poverty.

Findings from a study conducted in the slums of Mumbai show that traditional birth attendants are the most common health professionals to assist at home births and that the direct cost of a home birth was not substantially less than the cost of an institutional delivery in the public health sector (24). Customs and tradition, as opposed to cost, was the most common reason given for delivering at home (24). A study conducted in the slum of Indore showed that only half of the women who had given birth recently were well prepared for delivery and the possibility of an obstetric emergency (25). The same study showed that maternal literacy and use of ANC services were important predictors of birth preparedness. Untreated reproductive morbidities among women living in urban slums are common. Findings from a cross-sectional study conducted in Rajkot showed that as many as 57% were suffering from one or more reproductive morbidity and that only half of these women sought care for their complications (26).

Women living in rural parts of India are considered a vulnerable group in terms of maternal and reproductive health. In rural areas home births remain the most common practice, with only 29% of the deliveries taking place in a health facility (27). Results from a study based on data from the NFHS 1 and 2 on health-seeking behavior and institutional deliveries in rural areas showed that the influence of household wealth is stronger than the impact of geographical access (28). In a case-control study from rural Rajasthan, women from poor households were shown to be of nearly five times higher at risk of dying in pregnancy-related complication than women belonging to non-poor households (29). In rural Tamil Nadu, it was found that low use of contraception caused many women to undergo abortions to space or/and to limit births, and that a majority of the abortions identified in the study had been conducted by unqualified practitioners (30). A study using verbal autopsies to investigate pregnancy-related deaths in rural Rajasthan showed that 60% of the families of the deceased women had to borrow money to meet expenses for health care, which is suggested to have contributed to significant delays in seeking care (31). Finally, in rural Maharashtra a study showed that the capacity of the health system was low in terms of providing adequate iron supplements to women during pregnancy and that the public health system could not reassure availability of emergency obstetric care (32).

Over half (59%) of the pregnant women in India are anemic according to estimations based on data from the NFHS 3 (27). Furthermore, results from the same survey showed that anemia is common among all wealth quintiles in the society, 64.3% in the lowest wealth quintile and 46.1% in the highest quintile suffers from some type of anemia. A study conducted at health facilities in Karnataka found socioeconomic status to be the main contributing factor to high prevalence of anemia among pregnant women (33). Low knowledge in regards to anemia and health-seeking behavior is also associated with high prevalence of anemia (33). A study of severe anemia conducted at two maternity wards in New Delhi showed that 75.3% of the women the women suffering from severe anemia were admitted as an emergency, only 24.7% were diagnosed during a routine antenatal visit (34).

### Gender

Gender as a structural determinant of health operates through different intermediary determinants that influence the maternal and reproductive health of women and their access to care. In 2010 India was ranked as number 112 of 134 countries on the global gender gap index (35). Since the independence, the government of India has passed many laws to protect the rights of women. In general, however, implementation of many of these laws is weak. Only 55% of the women in India are literate compared to 78% of the men (28). Even in the younger age groups, 15–19 years, gender differences remain imbalanced with one in four women compared to one in ten men being illiterate (28). The position of women in the family and community is still weak. Data from the NFHS 3 conducted in 2005–2006 indicated that a majority of men think that husband and wife should make decisions jointly but that the husband should have the final word (36). This has implications for the health-seeking behavior of women, who may be dependent on their husband's permission to access health services.

In India, there is an association between the use of adequate prenatal, delivery and postnatal care and women's autonomy (37). The quality of family relationships and type of household is also associated with access to maternal and reproductive health care. A study using data from the NFHS 2 showed that women living in joint households (couple live together with the parents of the husband) were less likely than women living in nuclear households (households composed of husband, wife and children) to report use of contraceptives and less likely to utilize ANC. Women living in joint households where in-laws were present were less likely to either deliver in a health facility or in the presence of a skilled birth attendant (38). Data from the Women's Reproductive History Survey from 2002 revealed that women with better marital relationships and those who lived in nuclear households were more likely to use ANC services and to have an institutional delivery than others, while women living in joint families and who had better relationship with their in-laws were more likely to use ANC services (39). A majority of mothers-in-law in a qualitative study conducted in rural Madhya Pradesh was of the opinion that they should make decisions with regard to the use of sterilization among the wife(s) of their son(s) and that this decision often was based on the number of grandsons borne (40).

Domestic violence is a kind of gender-based violence. Several associations between domestic violence and women's reproductive health can be found in the context of India. One study, based on a follow-up of the NFHS 2 conducted in rural parts of Bihar, Jharkhand, Maharashtra, and Tamil Nadu, showed that women who experienced physical violence during pregnancy were less likely to receive ANC compared to women who did not experience physical violence (41). Women have also been shown to experience sexual violence in the form of marital rape during pregnancy (42). There is also an association between domestic violence and unintended pregnancy. A study based on data from the NFHS show that women who had experienced physical violence from their husbands were 47% more likely to report an unintended pregnancy (43). In Bihar, Jharkhand, Maharashtra and Tamil Nadu, it has been shown that women who experienced domestic violence from their husbands were less likely to have access to contraceptives (44). A qualitative study conducted in rural Maharashtra shows that non-use of contraceptives can partly be explained by the refusal of the husband, a decision often influenced by the opinion of the mother-in-law, and that the tension that may arise when husband and wife have different opinions on the use or non-use of contraceptives is seen in this community as a catalyst of domestic violence (45).

Gender norms have also been shown to influence attitudes toward the use of contraceptives and women's ability to make decisions on family planning. A qualitative study conducted in the western parts of India showed how women publicly were reluctant to acknowledge awareness and use of modern contraceptives and described the use of contraceptives, other than sterilization, as socially unacceptable (46). A study from rural West Bengal using both quantitative and qualitative methods showed how patriarchal structures influence women's use of contraceptives, especially those women who married at a young age (47). A qualitative study conducted in Maharashtra indicated restricted access to contraceptives, where issues related to reproductive health were considered to be a ‘women's issue’ and not commonly discussed between spouses but that it was the husband that made decisions in relation to heath care (48). A cross-sectional study conducted in a clinical setting in New Delhi also showed that reason for seeking abortion care were unplanned pregnancies (32.8%), inadequate income (24.6%), family complete (20.3%), and contraceptive failure (22.3%) (49). The study concludes that women do not only face barriers such as limited education and poverty, but they also face barriers due to lack of control of their reproductive intentions. Low use of contraceptives and gender norms that prevent married women from refusing their husband's sexual demands was shown in a study from Tamil Nadu to lead to unplanned pregnancies and a subsequent need for abortion (50).

Results from a qualitative study conducted on women seeking emergency obstetric care in Karnataka showed that maternal deaths are underreported, and not reviewed, and that ANC and institutional delivery are not linked to postpartum or emergency obstetric care (51). The results from this study indicates that a weak health system, including weak information systems, discontinuity of care, unsupported health workers and limited referral and accountability mechanisms, has implications for the ability to prevent maternal mortality among women seeking care during delivery in this setting.

### Education

Seventy-four percent of the Indian population is literate. Increased literacy rates are highlighted by the Indian Government as key in reducing inequities in India. Literacy among females rose from 53.7% in 2001 to 65% in 2011, a more rapid progress than the progress seen among men, where the literacy rate increased from 75.3 to 82.1% (52). Female literacy rates differ between states: from 92% in Kerala to 52.7% in Rajasthan (52).

The literature indicates a strong association between education and use of reproductive health services such as family planning and ANC. The NFHS 3 (28) indicated that the fertility rate among women with no education was 3.55, compared to 1.8 among women with 12 years or more education. Further, 29% of women with no education received at least one ANC visit as opposed to 88% among women with 12 years or more of complete education.

Other studies have found similar associations. A study from Madhya Pradesh showed that the odds of using ANC, skilled birth attendants, and postnatal care are significantly higher for women with secondary education and above compared to illiterate women (53). A large study from Uttar Pradesh looking at family planning among the urban poor showed that less educated women (1–11 years) living in both slum and non-slum areas are more likely to be sterilized, less likely to use other modern contraceptives, and more likely to have an unmet demand for family planning than more educated women (+12 years of education) (54). A study conducted based on data drawn from the NFHS 2 found that women with higher education (middle school and higher) were more likely to be using contraceptives (55). A cross-sectional study conducted in rural Punjab showed that 65.9% of the non-educated women, 78.1% of women with primary education, and 80% of the women with high school education were using contraceptives (56). Among illiterate women and women with primary education sterilization is the most common contraceptive method, while among women with high school education condoms were the most common (56). A study from New Delhi showed that illiterate women use contraceptives less compared to more educated women and when they do they prefer permanent methods of contraceptives (22). Level of education has also been shown to be associated with anemia, with less educated women being more likely to be anemic (57). A cross-sectional analysis using data for the state of Maharashtra from the NFHS 2 showed that level of education influences place of delivery, where 55.9% of women with primary education or less delivered at home compared to 25% among women with secondary education (58). The same study showed that there was not a wide difference in utilization of institutional delivery conducted at a public facility (28.8% among women with primary education or less and 34.7% among women with secondary education), but there was a considerable difference in use of private facility for an institutional delivery (15.3% among women with primary education or less and 40.3% among women with secondary education).

### Adolescence

Age can also be a source of health inequity. In India, children and adolescents (defined by the WHO as a person between 10 and 19 years of age) seem to have inequitable access to reproductive health. Pre-marital sexual relationships are widely discouraged in the Indian context and adolescent reproductive health remains taboo. There are few studies on the topic of adolescent reproductive health and national health surveys do not include non-married women when collecting data on women's maternal and reproductive health and utilization of health care. Some insights can be found in the subnational survey Youth in India: Situation and Needs conducted in 2006–2020 (59). The survey reports that 19% of men and 9% of women between 15–24 years had experience from a pre-marital romantic relationship, most of which included some form of physical intimacy. Further, two-fifths of the men and one-quarter of the women who reported to have experience from pre-marital romantic relationships had also engaged in a sexual relationship with the romantic partner (59). No large differences were found between adolescents living in rural areas compared to adolescents living in urban areas. Adolescents engaged in pre-marital sexual relationships reported low use of condoms: only 13% of the men and 3% of the women reported that they always used a condom (59). A survey conducted in Bihar and Jharkhand showed that unmarried young women experienced difficulties in accessing timely abortion care due to late recognition of pregnancy and difficulties in accessing health care (60, 61).

Child marriage remains a common practice in India, despite its illegality. Data from the NFHS 3 conducted in 2006–2007 show that among women in the age group of 20–24, 18% married before the age of 15, and 47% were married before the legal age of 18 (27). Moreover, in the lowest wealth quintile, as many as 78% of women was married before the age of 18. Young age at marriage has implications for women's reproductive health. It is associated with low use of contraceptives, unmet demand for spacing methods (62), with high fertility and multiple unwanted pregnancies (63, 64), and with poor obstetric outcomes (65). A large study looking at married adolescents (15–19 years) living in rural India showed low levels of full coverage of ANC (i.e. those having 3 ANC visits, IFA and TT2/booster) (14%), of skilled attendants at birth (46%), and of postnatal care (35%) (66). Women who were married before the age of 18 were more likely to have experienced physical and sexual domestic violence than women married after the age of 18 (67). Young age at marriage has also been shown to be associated with low use of ANC (68) and delivery care (67) among married adolescent pregnant women in India. A hospital based study conducted in Kolkata showed that teenage pregnancies were associated with more adverse complications, such as preterm births and stillbirths compared to adult mothers (69) and a study from Rajasthan showed that pregnant adolescent mothers were as two and half times higher at risk of dying from pregnancy-related complications than adults (29). Finally, young age at marriage was shown to be associated with high levels of anemia during pregnancy in a study in Maharashtra (70).

### Social class

#### Scheduled castes and scheduled tribes

Even though officially abandoned, the caste system is still apparent in the Indian society. In India the stratification of social class (caste) is one of the strongest social determinants of health (71). Furthermore, caste has been shown to be the most appropriate household characteristic for identifying poor and disadvantaged households. The summative term ‘socially backward classes’ (SBC) is commonly used in India to describe some of the most socially disadvantaged groups and includes the scheduled castes (SC) and scheduled tribes (ST) and other backward caste (OBC). They are not only distinguished by economic poverty but also by their marginalization and seclusion from the rest of the society, having different traditions and living in the most economically disadvantaged areas (71, 72).

Being a member of an OBC in India is associated with lower use of reproductive health services and poorer maternal health outcomes. According to the NFHS 3, the likelihood of receiving any type of ANC is lowest among women belonging to SC or ST. Only 18% of the births among these women are conducted at a health facility, compared to 51% among women who do not belong to SC, ST, or any OBC. These results are supported by state-level studies. For example, a study from Jharkhand showed that women who belong to a tribal caste are less likely to have received ANC, more likely to deliver at home, and less likely to have received a postnatal check-up (73). A study conducted in Uttar Pradesh showed that women belonging to SC or ST were less likely to obtain ANC and less likely to be assisted by a skilled birth attendant, although caste did not seem to have an impact on the use of iron folic acid supplement (74). A large study covering most parts of India showed that the odds of using skilled birth attendants at birth were considerably lower among women belonging to tribal castes than women belonging to non-tribal groups (75). Similar results from Jharkhand found that home deliveries are common in this state but that there are large differences between ST and other groups, 94% among women from a ST compared to 69% of the non-tribal groups (76). A study from Rajasthan looking at pregnancy-related deaths using verbal autopsies found that although only 37% of the women from the study sample belonged to the SC and ST, as much as 74% of the maternal deaths occurred among women belonging to these groups (31).

There are also several studies showing that contraceptive use is low among women belonging to tribal and SC. A study conducted on family planning among two ST in West Bengal showed that awareness of contraceptives methods other than sterilization was low and that the among women using contraceptives 97.6% belonging to the Lodhas tribe and 73% belonging to the Santal tribes were using sterilization (77). Similar results can be found in a study of two tribes in Andhra Pradesh where 75.6% were not using any contraceptive method at all and that permanent birth control (sterilization) was the most common type of contraceptive used (78). A study from Rajasthan showed that sterilization is the most commonly known and used contraceptive method among women belonging to tribal castes (79). Findings from a study from rural areas of Meghalaya showed that high levels of awareness of contraceptives may not increase the level of utilization amongst members of tribal castes (80).

#### Muslim community

The largest minority religion in India is Islam and the Muslim population is estimated to be around 138 million. A large proportion of the Muslim population is found in rural areas of West Bengal, Bihar, Maharashtra, and Uttar Pradesh. Religion in India overlaps with the caste system and 40.7% of the Muslim community belong to the group ‘other backwards castes’ (81). Forty-three percent of the Muslims live under the official poverty line and literacy rates among this group are lower than the national average. There is an under-representation of Muslims holding governmental and political positions in society. This review found only one article that was focused specifically on the Muslim population. Some of the studies include data in regards to religion but do not further explore the association between inequity in terms of maternal and reproductive health and belonging to a Muslim community. Below we present findings relevant for the Muslim population nested within studies where the primary focus is on another equity variable.

A study based on data from the NFHS 3 showed that the relative odds that a Muslim couple had concordant desired waiting time were only 42% of the odds that a Hindu couple had concordant desires (13). A study conducted in rural Punjab found that contraceptive use rate was 61.2% among Muslims, 72.8% among Hindus, and 82.1% among Sikhs (56). In a study looking at family planning among urban women in Uttar Pradesh, Muslim women were found to be less likely to be sterilized and less likely to be using contraceptives than non-Muslim women (54). A study looking at differences in use of reproductive health care among poor and non-poor groups in urban areas of India found that the difference between the poor and the non-poor are larger among Muslims than among non-Muslims (22). Compared to other religious groups, Muslim women were found to have significantly lower odds of being assisted by a trained birth attendant in a study conducted on data from the NFHS 3 (20). Married adolescent Muslim women (and women belonging to other religions) were found to be less likely to be using safe deliveries compared with Hindu women in a study also based on data drawn from the NFHS 3 (68) and a study using the same data set focused on married adolescent residents in rural areas also came to the conclusion that married adolescent Muslim women were less likely than married adolescent Hindu women to have a safe delivery (66). A study looking at factors influencing the use of maternal health care service in Madhya Pradesh found that Muslim women, and women belonging to non-ST, were more likely to receive ANC than other groups of women (53). Muslim women were found to be more likely to seek care for postpartum morbidities from formal providers as compared to Hindu women in a study conducted in rural West Bengal (82). An ethnographic study conducted in a rural Muslim village in Uttar Pradesh on perceptions and experiences in regards to institutional deliveries, showed how widespread mistrust toward the public health system, traditional practices in regards to the use of contraceptives and home deliveries, and poverty, interact in causing barriers for women to access skilled assistant at birth for Muslim women in this community (83). A survey conducted in a slum community in Mumbai with 85% of the population being Muslim found that one in four women reported abuse from in-laws during pregnancy (84). The survey was followed by a qualitative component which showed that there might be an association between abuse from in-laws and the sex of the baby in this community (84).

## Discussion

In the last few decades, Indian society has experienced rapid economic growth. However, large proportions of the population have not benefited from this progress. Balarajan et al. (9) describe how insufficient allocation of public spending on health between rural and urban areas and between preventative and curative services; access to quality care among the poor; and high out-of-the pocket expenditures act as key barriers to increased equitable access to health care in the setting of India. The findings from this review adds to their analysis by pinpointing the populations that seem to be systematically and consistently disadvantaged in Indian society in terms of access to and use of maternal and reproductive health services. These populations – as identified in the published literature – are poor women, the poorly educated, adolescents, Muslims and members of SC and ST. Below, we refer back to the CSDH framework in a discussion on the findings from this review. How these findings can be utilized to identify entry points for the development of policy on maternal and reproductive health in the setting of India is further developed in the section on policy implications further down in this article.

Social power is a central concept in the CSDH framework in explaining the dynamics of social stratification. The framework is influenced by previous research, especially a model developed by Diderichsen (85). This model accentuates how the context creates social stratifications that assign different social positions to individuals. These social positions influence the exposure and vulnerability of individuals in terms of conditions causing ill-health. This review found that in the Indian context, economic status, gender, social class, education, and age are important stratifiers in understanding what influences access to and use of maternal and reproductive health care. The social power that individual women possess is influenced by their economic position, gender norms, and social class and this in turn affects their access to and use of health care.

Progress toward reaching MDG 5 in India is unevenly distributed between wealth quintiles, with women belonging to the lowest wealth quintiles falling behind (11, 12). Economic status is shown in this review to intermediate through society and influence access to contraceptives and adequate care during pregnancy and delivery for women belonging to poor households. The findings from this review suggest that poverty causes inequity all over India, and that residency is less important than economic status. Both the population living in urban slums and the poor living in rural areas have less access to maternal and reproductive health care compared to the non-poor living in the same areas (20, 28).

In addition to economic status, the stratification of caste is one of the strongest social determinants of health in the setting of India (71). Caste and economic status are closely interlinked, with women from marginalized caste groups often also being poor. Many of the articles discussing caste reviewed in this study are focused on wealth when studying inequity in terms of access to maternal and reproductive health for this group. However, it is important to recognize that economic wealth does not always neutralize the effects of belonging to a SC or ST. The importance of caste as a determinant of inequity in health has been shown in other studies conducted in India. A study conducted in Kerala concluded that caste-based inequity in household health expenditure reflects unequal access to general health care by different caste groups (71). A review of social exclusion, caste, and health concluded that the health status and health care-seeking behavior of SC and ST provides an indication of both their social exclusion as well as the linkage between poverty and health for this population (72). The findings from this review show that women belonging to these groups are less likely to be assisted by a skilled birth attendant (75) and there is an unmet demand for modern contraceptives among this group (77, 78). In the review presented in this paper, we indicate that caste may influence not only access to but also quality of care received. This however, seems to be an understudied topic that needs to be further explored.

Indian society is largely stratified by gender and patrilineal descent and women's autonomy in terms of decision-making, mobility and access to and control over resources is constrained (86). Gender equity, including female literacy, education and decision power, is closely linked to reproductive health (87). Low economic status and gender biases are often considered to be interlinked, however, Sen and Iyer (88) and Iyer et al. (89) has shown that ‘pure gender bias’ exists in access to health care in India. Findings from this review show that women's position in the household (38, 39) and in the community (46, 47, 50) influences women's access to contraceptives, use of abortion services, and health care-seeking behavior during pregnancy and delivery. Gender norms are closely linked to adolescent's sexual and reproductive health. Pre-marital sexual relations are widely discouraged in the Indian context, and this review found studies showing that adolescents face barriers in accessing contraceptives and reproductive health services. Unmarried adolescents and youth are not included in the large surveys on population health in India and are often left out when studying maternal and reproductive health in the India. More focus on adolescent's sexual and reproductive health is needed to target this group. The common practice of child marriage can also be seen as a reflection of gender norms in the Indian society. Almost half of the women between the ages of 20–24 years were married before the age of 18 (27). Child marriage often means that the woman's autonomy to make decision on her sexual and reproductive health is limited. The findings from this review shows that young age at marriage is associated with low use of contraceptives and unmet need of spacing methods, low use of maternal health care and higher risk of suffering from maternal mortality (62–64, 66). The common practice of child marriage and its implications for maternal and reproductive health needs to be acknowledged in the setting of India and efforts needs to be taken to implement the law that forbids marriage before the age of 18. In summary, gender norms restrict the social power of women in the setting of India, influencing the access to maternal and reproductive health care, which is likely to have an impact on maternal and reproductive health outcomes.

In India, one in three women is illiterate (52). The association between education and maternal and reproductive health found in this review was mostly related to family planning. Less educated women are more likely to have an unmet demand of contraceptives and more likely to be sterilized than more educated women (21, 54–56).

One of the major findings that emerged from this review is that structural determinants of inequity relevant for understanding maternal and reproductive health in the setting of India are closely interlinked and difficult to separate from each other. This linkage is supported by the CSDH framework. The framework emphasizes the close relationship between the context and structural determinants, and between the different structural determinants, to determine inequity. When targeting inequity in terms of access to maternal and reproductive health care, economic status, caste and gender need to be considered jointly. This conclusion is supported by findings from a large systematic review looking at the impact of interventions on maternal and reproductive health in poor settings (90). The review conducted by Nyamtema et al. shows that there are no single magic bullets that can reduce maternal mortality. Inequity due to poverty is an important factor but progress toward improved maternal and reproductive health is dependent on a wide range of interventions. More studies are needed to understand how economic status, gender and caste jointly cause inequitable access to maternal and reproductive health in the context of India, and interventions targeting marginalized and vulnerable groups needs to take all three factors into consideration. However, India is a large country and there large differences between states both in terms of progress among marginalized groups and in terms of interventions implemented to reach these groups. More studies focused on specific states are needed to provide a contextualized analysis of the situation and to provide insights on how to design policy.

## Limitations

This review has several limitations. The search strategy was carefully developed but it is possible that articles relevant for the objective of this study were not identified using the search terms for this study. The objective of this review was to describe the current situation and did not include intervention studies. The intervention studies may have provided information relevant when describing structural determinants of equity in regards to maternal and reproductive health in India. We are currently conducting a systematic review of interventions proven to be effective in reducing inequity in maternal and child health globally, including in India. It is also possible that there are other structural determinants of equity relevant to maternal and reproductive health in India but these have not been studied and/or published in peer-reviewed journals. Moreover, some of the themes we identified in the literature are clearly well-studied in India (such as economic status and gender), whereas others are less so (education, age, and social class). In fact, the studies on education, adolescence and to some degree social class are often nested within studies of economic status and/or gender. This points to a general lack of published research on these topics that limit our understanding. Most studies identified were based on quantitative data. Such studies do not provide explanations, for example, for inequity based on economic disparities. More qualitative research is needed on this topic in India.

## Policy implications

This review has shown that it is not sufficient to speak of achieving MDG 5 in India as a whole. The differences between states, places of residence, age groups, social status and economic background are simply too great in India to allow such generalizations. A very focused campaign of action delivery services to the most disadvantaged, to reduce inequities in health, particularly in maternal and reproductive health, will be necessary at the national, state, and district levels to have a significant impact on the most disadvantaged populations. In addition, due to the wide disparities between and within states, which are often caused by varying policies and programs, health infrastructural shortcomings and governance challenges, analysis of inequities in maternal health care need to be undertaken at the state and district level. This also calls for additional research focusing on specific geographical areas or a specific group within the population. Especially qualitative research is needed to answer questions on how social determinants influence access to and use of maternal and reproductive health in a specific setting or among a specific community. Disparities between regions and subpopulations also emphasize the need for context sensitive policies and programs. It is clear that one-size will not fit all and especially the poor and vulnerable will be left out. How social determinants interplay in a specific context influencing access to and use of maternal and reproductive health needs to be carefully considered when designing and implementing policy and interventions.

Our review identified several key populations that are disadvantaged in terms of access to and use of maternal and reproductive health services that need to be addressed by policymakers and programmers across India. Some of these populations are well-known but still under-served, such as the poor and the socially backward castes. Although the Indian government has attempted to rectify some of these inequities with, for example, conditional cash transfers to encourage women to give birth in institutions with skilled birth attendants under Janani Suraksha Yojana (JSY), the measures are not sufficient to reach the most disadvantaged population (91). Also, the quality of care under JSY is suspect and hence its impact on outcomes such as MMR and IMR will be doubtful.

This review shows that the cost for maternal health care is a significant burden on households among the poor and socially backwards castes. Interventions targeting the cost for transportation and loss of income are needed, as well as intervention reducing the burden of out-of-pocket payment for care. The mechanisms through which economic status causes barriers to care may differ between geographical areas and it is likely that different strategies are needed to address inequity due to poverty depending on residency. There is also a need for strengthening the health system capacity to provide care to this fragment of the population, both in terms of referral systems and the quality of care.

This study also highlights a disadvantaged population in terms of maternal and reproductive health that has not received sufficient attention: adolescents. Adolescents represent 20% of the population in India (92), and without appropriate attention to sexual and reproductive morbidity and mortality in this age group, India will not be likely to reach equity in achievement of MDG5. The association between child marriage and poor maternal and reproductive health outcomes is evident in the findings from this review and India needs to increase the efforts to implement the law forbidding marriage before the age of 18. Further, the non-married adolescents that presently is non-existing in surveys and studies needs to be being acknowledged and included.

This review shows that there is an overlap in how economic status; gender and social status interact when influencing use of and access to maternal and reproductive health care. This makes it important to consider all these in relation to each other when designing policies and programs. For example, a program addressing economic barriers should also consider social status and gender since it likely that intermediary determinants caused by theses are closely linked to the intermediary determinants that the program is targeting.

The government of India and state governments should consider following specific steps to reduce the inequity gap. There has to be substantial improvement of services in terms of capacity and quality in the public system where the poor and vulnerable live. Maternal and health care needs to be provided close to their homes, thus reducing the distance barrier. The Indian Government should develop and annually publish special rates of infant mortality rate, maternal mortality rate, and still birth rate for marginalized groups, such as the poor and tribals, through an expansion of the Sample Registration System already implemented. One of the reasons that services are poor in remote rural and tribal areas is difficulties in recruiting doctor or health workers to these areas, thus creating barriers to service delivery or health education. This can be reduced by creating a special cadre or force of doctors, nurses, and health staff to run the health centers in remote and poor areas. The staff recruited will have to be specially selected based on commitment and attitude for serving the poor and marginalized. Furthermore, incentives, in the form of high salaries and other benefits such as better housing, need to be provided to recruited staff. A special strategy in regards to services during delivery will be needed to reach out the most vulnerable and marginalized.

## Conclusion

India is making progress toward reduced maternal mortality and improved access to reproductive health care. However, evidence shows that the progress made is uneven and inequitable. The objective of this review was to describe the evidence in terms of structural determinants of maternal and reproductive health in India, and how these causes affect access to care. The collective picture that is presented in this paper is that structural determinants prevent reduced maternal mortality and increased access to reproductive health for women belonging to disadvantaged populations. Interventions that target maternal mortality and increased access to reproductive health care need to take into account how these structural determinants operate in the Indian society and how this may influence access to health care for certain groups of women.

## Appendix 1. Search terms

**#1: Equity**

**MESH terms:** Healthcare Disparities; Health Status Disparities; Universal Coverage; Vulnerable Populations; Poverty; Rural Population; Residential Mobility; Emigration and Immigration; Minority Groups; Religion; Hinduism; Islam; Christianity; Ethnic Groups; Adolescent; Social Class; Socioeconomic Factors; Insurance Coverage; Health Resources; Health Manpower; Equipment and Supplies, Cost, Gender

**Free terms:** Equity; inequity; inequities; equality; inequality; inequalities; disparity; disparities; universal coverage; disadvantaged group; disadvantaged groups; disadvantaged population; disadvantaged populations; vulnerable population; vulnerable populations; sensitive population; sensitive populations; poor; poverty; indigene; indigent; indigency; self-employed; informal sector; informal sectors; low-income; unemployed; slum; rural; urban; agricultural worker; agricultural workers; agricultural labor; agricultural labors; farmer; farmers; farmworker; farmworkers; migration; migrations; migrant; migrants; emigrant; emigrants; immigrant; immigrants; minority; minorities; ethnicity; ethic; religion; religious; tribal; scheduled; hindu; islam; muslim; muslims; Christian; Christianity; adolescent; adolescents; youth; youths; teen; teens; teenager; teenagers; social class; social classes; caste; castes; socioeconomic; financial constraints; geographical constraints; social norms; health workers attitude; health workers attitudes; health resource; health resources; human resource; human resources; infrastructure; transportation; supplies; equipments; devices; education; discriminatory; discrimination; uninsured; insured; insurance status; insurance coverage

**#2: Maternal health**

**MESH terms**: Maternal Health Services; Maternal Welfare; Maternal Mortality; Contraception; Family Planning Services; Pregnancy; Abortion, Induced; Abortion, Spontaneous; Prenatal Care; Delivery, Obstetric; Postnatal Care

**Free terms:** Maternal health care; maternal healthcare; maternal health; maternal services; maternal mortality; maternal mortalities; maternal morbidity; contraception; birth control; fertilization control; family planning; planned pregnancies; pregnancy; gestation; abortion; pre-natal care; pre-natal visit; pre-natal visits; prenatal care; prenatal visit; prenatal visits; antenatal care; antenatal visit; antenatal visits; obstetric delivery; obstetric deliveries; delivery care; facility-based delivery; skilled birth attendance; postnatal care; post-natal care; postpartum program; postpartum programs; safe motherhood

**#3: India**, Indian

## Appendix 2. List of references

**Table:**

Title, author(s)	Year	Inequity variable	Sample	Study location	Design	Outcome
Agrawal et al. Birth preparedness and complication readiness among slum women in Indore, India.	2010	Economic status	312 mothers of infants 2–4 months of age, living in urban slum.	Indore, Madhya Pradesh	Cross-sectional study. Multivariate analysis using binary logistic regression was carried out.	47.8% of the women were well-prepared. Of those not prepared (30.4%), lack of perceived need, economic constraints, and lack of faith in TBA/public health system was given as reasons.
Bonu et al. Incidence and correlates of ‘catastrophic’ maternal health care expenditure in India.	2009	Economic status	6,879 women, aged 15–49, pregnant during the 365 days prior to the survey.	India	The study uses data from the 60th round of the National Sample Survey (NSS) of India (2004).	73% and 64% obtained ANC and PNC, respectively, and 43% had an institutional delivery. All the households from the poorest decile and 99% of the second poorest decile paid more than 40% of their capacity to pay.
Hazarika. Women's reproductive health in slum populations in India: Evidence from NFHS 3.	2010	Economic status	A subsample of 4,827 women (2,420 living in slum areas, 2,407 living in non-slum areas).	8 cities in India, including the major cities	Data were drawn from the NFHS 3. In the survey, data on urban slum were collected from 8 cities in India.	The odds of using modern contraception were significantly lower among slum residents than non-slum residents. Factors associated with use was age, level of education, parity, and employment status, while factors such as religion, economic status, financial autonomy, partner's education/occupation was not found significant. Women in slums were less likely to complete 3 ANC; however, the result was not statistically significant. Skilled attendance at birth was significantly associated with age, level of education, economic status, parity, working status, financial autonomy, and prior ANCs. Partner education/occupation was not associated with SBA.
Kumar and Mohanty. Intra-urban differentials in the utilization of reproductive health care in India, 1992–2006.	2011	Economic status	The urban sample from NFHS 1 covered 28,822 households and 27,534 women, and the urban sample of NFHS 3 covered 50,236 households and 56,961 women.	Major states in India	Data were drawn from the first and third rounds of NFHSs.	While the non-poor/poor gap in antenatal care and medical assistance at delivery remained large over the years, the gap in contraceptive use has narrowed down cutting across states. After adjusting for confounders, household poverty was found to be a significant barrier in the utilization of reproductive health care services across the states.
Skordis-Worall et al. Maternal and neonatal health expenditure in Mumbai slums (India): A cross-sectional study.	2011	Economic status	1,200 slum residents in Mumbai	Mumbai	The study uses expenditure data for maternal and neonatal care, analyzed by socioeconomic status, calculating a Kakwani Index for a range of spending categories. Catastrophic health spending was also calculated both with and without adjustment for coping strategies.	A higher proportion of respondents spent catastrophically on maternal health care. Lower SES was associated with higher proportion of informal payments. Indirect health expenditure was found to be (weakly) regressive since the poorest were more likely to use wage income to meet health expenses, while the less poor were likely to use savings.
Das et al. Prospective study of determinants and costs of home births in Mumbai slums.	2010	Economic status	Women living in slum areas. Interviews were conducted 6 weeks after giving birth.	Mumbai, Maharashtra	Data were drawn from a key informant surveillance system used to identify birth prospectively in 48 slum communities in six wards of Mumbai, covering a population of 280,000, data were also collected using a closed questionnaire.	The odds of home birth increased with literacy, parity, socioeconomic poverty, poorer housing, lack of water supply, population transience, and hazardous location.
Kesterton et al. Institutional delivery in rural India: the relative importance of accessibility and economic status.	2010	Economic status	A large sample of births taken place in rural areas (not sure how many) was drawn from the data from NFHS 1 and 2.	Rural areas of India	Data are drawn from NFHS 1 and 2.	The adjusted results show that the influence of household wealth is stronger than that of geographical access. Regional differences are strikingly large and the low probability of institutional delivery in the North cannot be explained by disparities of access, wealth of education.
Patra et al. Clinical profile of women with severe anemia in the third trimester of pregnancy.	2010	Economic status	130 pregnant women admitted to Lady Hardinger Medical College and the Smt. S.K. Hospital in New Delhi due to severe anemia.	New Delhi, clinical setting	No description of method.	12% of the women were teenagers. 43% of the women had a birth interval less than 1 year. 75% were illiterate and 84.6% lived in households with low socioeconomic status (classified as per Kupuswamy's scale). 73.8% had not received any iron or folic acid supplements. The majority, 75.3%, were admitted as an emergency but a significant 24.7% were diagnosed during a routine antenatal visit.
Bhanderi and Kannan. Untreated reproductive morbidities among ever-married women of slums of Rajkot, Gujarat: the role of class, distance, provider attitudes, and perceived quality of care.	2010	Economic status	593 women (aged 15–49) that had experienced maternal morbidities, drawn from a sample of 1,046.	Rajkot, Gujarat	A community-based, cross-sectional study. Data were collected using structured interviews.	After controlling for other variable: SLI, belonging to scheduled castes/tribes, distance from maternity health facility and duration of illness greater than 1 year were all found to be significantly associated with un treated reproductive morbidity.
Jeffery and Jeffery. Only when the boat has started sinking: A maternal death in rural north India.	2010	Economic status	People living in the village of Jhakri.	Bijnor district, north-western Utter Pradesh (rural)	Ethnographic design was used. Data collection was ongoing for a longer period of time (2002–2005).	
Gupta et al. Maternal mortality ratio and predictors of maternal deaths in selected desert districts in Rajasthan: a community-based survey and case control study.	2010	Economic status	25,926 households were surveyed in 411 villages, 32 maternal deaths and 6,165 live births were identified.	4 districts in Rajasthan (Bikaner, Barmer, Jaisalmer, Jodhpur)	The study has two major components: a community-based household survey and a case–control study with cases and controls sampled from the same population. A total of 32 maternal deaths and 6,165 live births were identified. The group of women who died during pregnancy or delivery (cases) was compared with the group of women who gave birth and survived (controls).	MMR was found to be 519 per 100,000 live births. The cases and controls were fairly comparable on sociodemographic characteristics. There were significant differences in poverty status, first pregnancy, problems during pregnancy and delivery, and place of delivery between the two groups.
Pathak et al. Economic inequalities in maternal health care: prenatal care and skilled birth attendance in India, 1992–2006.	2010	Economic status	90,000 households in each survey.	All India	Data from NFHS 1, 2, and 3 were used. Analysis of data was carried out for rural, urban and combined data. Estimations of prevalence of prenatal care and skilled birth attendance by economic status and residence were done.	Increase in PNC between 92–06 among non-poor 23.5%–35.3% and among poor 6.1%–6.2%. Large differences between progresses in different states, however, across all states PNC among poor remained substantially lower than among non-poor. Use of SBA across states also showed considerable lower use among poor than non-poor. SBA also remained lower among poor across a rural-urban spectrum.
Mohanty and Pathak. Rich-poor gap in utilization of reproductive and child health services in India, 1992–2005.	2009	Economic status	In Uttar Pradesh 8,338 (NFHS1), 7,590 (NFHS2), and 10,026 (NFHS3) households were included. The figures for Maharashtra were 4,063, 5,830, and 8,315.	Uttar Pradesh and Maharashtra	Data were drawn from three rounds of NFHS. Bivariate analysis was carried out to understand the differentials in health care utilization by wealth quintiles.	The results show wide disparities in utilization of maternal and reproductive services, largely to the disadvantage of the poor. The use of SBA and ANC remained low among the poor during this period. However, contraceptive use increased relatively faster among the poor.
Rani et al. Differentials in the quality of antenatal care in India.	2008	Economic status	840 women from south India and 2,970 from north India.	4 north Indian states (Bihar, Madhya Pradesh, Rajasthan and Uttar Pradesh) and 4 south Indian states (Andhra Pradesh, Karnataka, Kerala and Tamil Nadu)	Data from the NFHS 2 was used. Bivariate analysis and multivariate linear regression models were used.	Significant socio-economic differentials in the quality of care were evident in both north and south India, but were more glaring in the north. A significant positive relationship was observed between quality and utilization of antenatal care in rural areas.
Dhar et al. Quality of care, maternal attitude and common physician practices across the social-economic spectrum: a community survey.	2010	Economic status	50 women from a high-income area, 99 from a middle-income area, and 100 from a low-income area. In total 249 women.	South Delhi	Data were collected using questionnaires. Hemoglobin, blood pressure, weight and height were measured.	Substantial sections of the low-income population fail to meet some of the minimal national public health goals. Health care providers were unable to meet the national standards on minimal care during pregnancy and delivery in the poorer areas of the city, whereas this did not to seem to be a problem in the higher-income areas of the city.
Dhar et al. Direct cost of maternity-care services in South Delhi: A community survey.	2009	Economic status				
Varkey et al. The reality of unsafe abortions in a rural community in south India.	2000	Economic status	195 women	Rural Tamil Nadu	A community-based study with a cross-sectional design was carried out. Only the results from the cross-sectional study are presented in this article.	28% of the women had had an abortion. 84% of the women assumed that abortions were illegal in India. 23% thought that it was normal to have at least one abortion during their lifetime. Induced abortions were used both as a spacing method and as a way to limit family size. Abortions were often carried out using unmodern ways and a majority was conducted by untrained and un registered providers.
Singh and Becker. Concordance between partners in desired waiting time to birth for newlyweds in India.	2011	Economic status	883 newlywed (less than 12 months).	All India	Data are drawn from NFHS-3. Binary logistic regression was carried out.	65% of the couples have concordant desired waiting time. Couples belonging to the richer quintile had twice the odds as couples from the poorest quintile to have concordant desired waiting time to first birth.
Tuddenham et al. Care seeking for postpartum morbidities in Murshidabad, rural India.	2010	Economic status	929 mothers who reported have had a postpartum morbidity within 6 weeks after delivery.		Data were collected through a household survey in the Murshidabad district in 2008. Multinomial logistic regression was conducted.	5.8% of the women did not seek care, 49.2% sought care from informal providers and 45% sought care from formal providers. Women living in households where the head of the household had higher level of education the women were more likely to seek care from a formal provide than from an informal. Women who had experienced severe morbidity were associated with seeking care from a formal provider. Hindu women were found to be 39% less likely to seek care from formal provider as opposed to informal provider than Muslim women. Women who did not seek any care appear to be influenced by distance.
Gupta et al. Reproductive and child health inequalities in Chandigarh Union Territory of India.	2008	Economic status	1,200 households were included and 5,383 women aged 15–49 was interviewed.	Chandigarh Union Territory, North India	A household survey using standard multi-indicator cluster sampling method.	Coverage of maternal health service was lowest in slums compared to the rural and urban areas. Institutional deliveries were significantly low in slums compared to the urban and rural areas. Prevalence of contraception was higher in urban (73%) and rural areas (75%) compared to slums (53.4%).
Chaturvedi and Ranadive. Are we really making motherhood safe? A study of provision of iron supplements and emergency obstetric care in rural Maharashtra.	2007	Economic status	14 PHC, District health officers (DHOs), 3 rural hospitals.	Rural Maharashtra	Questionnaires were used for data collection.	During the year of 2006, when the data were collected, no PHC received any iron supplements despite demands of the DHOs. DHOs refer to a shortage within the district. No caesarean sections were conducted at the selected hospitals.
Jat et al. Factors affecting the use of maternal health: services in Madhya Pradesh state of India: a multilevel analysis.	2011	Economic status/education	15,782 ever married women aged 15–49.	Madhya Pradesh	Quantitative study. Sample drawn from the DLHS-3 conducted in 2007–2008. Multilevel logistic regression was carried out.	ANC: Household socio-economic status and mother's education were found to be the strongest individual-level factors related to the use of ANC. The odds of use of ANC among women with higher secondary and above education were 2.57 times higher than that of illiterate women. SKB: Mother's education, use of ANC and household SES were the strongest factors associated with skilled attendance at delivery. Women with higher secondary and above education were 2.35 times more likely to receive SKB compared to illiterate women. PNC: the odds of reporting the use of PNC among women with secondary and above education were 1.39 times higher than among those who were illiterate.
Kumar et al. Contraceptive use among low-income urban married women in India.	2011	Economic status/education	7,846 married women, aged 18–45, with low socioeconomic status.	Balak Ram Hospital, north of Delhi.	Quantitative study using questionnaires. Data collected over 2 years (2007–2009).	52% were using some form of contraception; most preferred a permanent method of contraception rather than using spacing methods. Level of education was shown to be associated with the use of any contraception and the use of spacing methods. Illiterate women were using less contraceptives and when used they preferred permanent methods compared to more educated women.
Noronha et al. Maternal risk factors and anemia in pregnancy: a prospective retrospective cohort study.	2010	Economic status/education	1,077 antenatal women (below 14 weeks of gestation) and 1,000 postnatal women.	Udupi district, Karnataka	A combination of prospective and retrospective cohort approach: the antenatal women were studied using a prospective approach and the postnatal women were studied using a retrospective approach. A standard questionnaire was used to obtain data on demographics and knowledge. The hemoglobin was estimated using the cyanmethemoglobin method.	50.14% of the women belong to the antenatal group and 53.7% of the women belonging to the postnatal group suffered from anemia. Low socioeconomic status was found to be the main contributing factor for higher prevalence of anemia.
Basu et al. Knowledge, attitude and practice of family planning among tribals.	2004	Social class	600 households with at least one ever married women in the age group 15–49.	Among the two tribal groups Santal and Lodha in the district of Midnapore, West Bengal.	Questionnaires were used. Descriptive statistics. Multiple regressions were not used.	Contraception was used to limit family size rather than as a spacing methods. (Sterilization was the most known and used method). The study found indications of that economic status could act as an incentive for limiting family size.
Bhasin and Nag. Demography of the tribal groups of Rajasthan: 5. Dynamics of family planning methods usage.	2007	Social class	900 nuclear families belonging to 661 households, Primary respondent was the ever married women in the household aged 15–49.	6 ST (Sahariya, Mina, Bhil, Kathodi, Damor, Garasia) spread over 6 districts in the state of Rajasthan.	Structured questionnaires and participant observation was used. Descriptive statistics were used. Each ST is presented individually and some comparisons are made between the different groups. No multiple regressions are conducted.	Sterilization is the most commonly known and used family planning method. Despite almost universal knowledge of at least on form of contraception (99%) the percentage of ever-users of any type of family planning method is quite low (47.8).
Lakshmi et al. Perceptions towards family planning - a study on tribal women from Andhra Pradesh.	2011	Social class	602 nursing mothers.	Sample drawn from two tribal groups, Savara and Jatapu, spread over 78 villages located in the district of Srikakulam, Andhra Pradesh.	Survey using questionnaires. Descriptive statistics. No regression was conducted.	The use of family planning methods are low in both groups, 22.5% of Savaras and 26.2% of Jatapus have adopted a method of family planning. And the majority of these, 98.5% among Savara and 85.2% of Jatapus, have adopted permanent birth control methods.
Hazarika. Factors that determine the use of skilled care during delivery in India: implications for achievement of MDG-5 targets.	2011	Social class	31,797 ever married women, aged 15–49	India.	Sample drawn from the NFHS 3. Bivariate and multivariate techniques were used.	The odds of using skilled delivery care were low in the two extreme age-groups (15–24, 45–49). Women living in rural areas are less likely than those living in urban areas to use SBA. Women with higher SES and higher education is more likely than women with lower SES and low education to use SBA. Muslim women and women from ST have significantly lower odds of using SBA than non-Muslim and non-ST.
Maiti et al. Health care and health among tribal women in Jharkhand: a situational analysis.	2005	Social class	1,614 ever married women aged 15–49, including 469 tribal women and 1,145 non-tribal women.	The state of Jharkhand	Quantitative study using a sample drawn from the NFHS II. Simple bivariate analysis is conducted and the result is presented in percentage.	ANC: One in every four tribal women undergoes ANC compared to 44% of the non-tribal women, 55% if ST do not receive a single dose of TT vaccine during pregnancy compared to 37% non-ST. DELIVERY: 92% (ST) compared to 78% (non-ST) gave birth at home, 5% (ST) compared to 17% (non-ST) were assisted by a trained person during delivery. POSTNATAL CARE: 11% (ST) compared to 19% (non-ST) were followed up by a check-up within two months of delivery. CONTRACEPTIVE USE: 15% (ST) compared to 31% (non-ST) of currently married women were using one or more method of contraception. ANEMIA: 75% (ST) compared to 64% (non-ST) are anemic.
Agrawal and Agrawal. To what extent are the indigenous women of Jharkhand, India living in disadvantageous conditions: findings India's National Family Health Survey.	2010	Social class	1,614 ever married women (469 women belonging to ST and 1,145 belonging to a non-ST group).	The state of Jharkhand	Sample drawn from the NFHS 2. Descriptive statistics, comparing health women belonging to ST with women belonging to non-ST group. No regression was made.	Several outcomes. Most interesting for our objective: Women belonging to ST are married at younger age than women belonging to non-ST group.
Raj et al. Abuse from in-laws during pregnancy and postpartum: qualitative and quantitative findings from low-income mothers of infants in Mumbai.	2011	Gender	1,070 women seeking care for their infants.	Mumbai	Qualitative data were collected through in-depth interviews and using grounded theory for the design of data collection and analysis. Quantitative data were collected in a survey and adjusted regression analysis was conducted.	1 in 4 women had experienced abuse from in-laws. The study suggests that the may be a link between the sex and the infant and abuse from in-laws.
Saroha et al. Caste and maternal health care service use among rural Hindu women in Maitha, Uttar Pradesh, India.	2008	Social class	482 Hindu women living in 29 villages and 11 hamlets of the Maitha block of Kanpur Dehat, UP, all had experiences from an abortion or a planned home delivery.	Rural Utter Pradesh	Data are drawn from the Morbidity and Performance Assessment (MAP) study, which is a population-based retrospective cross-sectional study. Correlation analysis was used to identify potential multicollinearity between caste and sociodemographic variables. Multivariable regression analysis was conducted to determine the odds of higher use among upper-caste women.	Maternal health care use among Hindu women in Maitha were low in both groups. The logistic regression analysis showed that upper-caste women were almost 3 times more likely to use ANC and 5 times more likely to be attended by a trained attendant at birth compared to lower-caste women and almost.
Iyengar et al. Pregnancy-related deaths in rural Rajasthan, India: Exploring causes, context, and care-seeking through verbal autopsy.	2009	Social class	160 deaths among women in reproductive age was investigated.	A block in southern Rajasthan, including 160 villages and a population of about 142,000.	Data were collected using pre-tested questionnaires.	Although only 37% of the population of the block belonged to SC or ST, 74% of maternal deaths were among these groups. 60% of the families had to borrow money to pay for care, which contributed to delays in care seeking or avoiding care. Three-fourths of the deaths in the study occurred at home and several women who sought care outside the home eventually died at home.
Deb. Knowledge, attitude and practices related to family planning methods among the Khasi Tribes of East Khasi hills Meghalaya.	2010	Social class	1,560 ever married Khasi women aged 15–49.	Meghalaya	Data were collected using both quantitative and qualitative methods. Analysis not described.	52.7% were using some form of family planning method. Only 4% of the women were not aware of any family planning methods. High levels of awareness of contraceptives may not increase the level of utilization amongst members of tribal castes.
Thind et al. Where to deliver? Analysis of choice of delivery location from a national survey in India.	2008	Social class/education	1,510 births was drawn from a sample of 5,391 ever married women, aged 15–49.	Maharashtra State, Western India	Cross-sectional study using data from the NFHS 2. Multinomial logistic regression analysis was conducted. Andersen Behavioral Model was used as conceptional framework.	Results from the multinominal logistic regression: women with higher birth order and those living in rural areas had greater odds of delivering at home compared to using public facilities; while increased maternal age, greater media exposure, and more than 3 ANC were associated with greater odds of delivery in a public facility. Compared to Hindu women, Muslim women had lesser odds of delivering at home. Additionally: the odds of home delivery decreased as the standard of living increased.
Mistry et al. Women's autonomy and pregnancy care in rural India: a contextual analysis.	2009	Gender	11,684 rural, married women that had given birth to at least one child and living in a rural area.	2,115 rural villages around India	Data were drawn from the NFHS 2. Three measures of care where included: adequate prenatal care utilization, delivery care and postnatal checkup. Women's autonomy was measured in three dimensions: decision-making autonomy, permission to go out, and financial autonomy. Multilevel logistic regression models were conducted.	SBA was least influenced by women autonomy; only financial autonomy was significantly associated with SBA. Decision-making autonomy was associated with the use of prenatal care and postnatal care. Permission to go out and financial autonomy was associated with both ANC and PNC. Regional differences in autonomy and care: in east India there was an association only between financial autonomy and the odds of having an institutional delivery: in south the women's autonomy was most consistently associated with care: and in the north autonomy was associated with ANC and PNC but not with SBA.
Saikia and Singh. Does type of household affect maternal health? Evidence from India.	2009	Gender	Sample of around 90,000 ever married women, aged 15–49.	26 states in India	Data were drawn from NFHS 2. Bivariate and multivariate analysis was conducted to examine the effect of type of household in regards to maternal health. Multinominal logistic regression was conducted to identify the determinants of contraceptive behavior and the utilization of ANC.	The multivariate analysis showed that: women living in joint households were less likely than women living in nuclear households to report use of contraception (even after adjusting for important socioeconomic and demographic characteristics): women living in joint households were less likely to access ANC compared to women living in nuclear households: women living in joint households with in-laws were less likely to have an institutional delivery of SBA compared to women living in joint families without in-laws of living in nuclear households.
Allendorf. The quality of family relationships and use of maternal health-care services in India.	2010	Gender	2,444 women, respondents were selected from all six regions of Madhya Pradesh, aged 15–39, and with at least one child.	Madhya Pradesh	Data are drawn from the 2002 Women's Reproductive Histories Survey (WRHS). Multivariate logistic regression is used to test whether women with higher-quality family relationships are more likely than others to use maternal health care services.	The link between the quality of marital relationship and use of maternal health care appears to be dependent on type of household (if the spouses live in a joint- and nuclear family). Among nuclear families, a high-quality relationship increase use of antenatal care and institutional delivery. In joint families, a good relationship with in-laws increases use of ANC and institutional delivery.
Koski et al. Physical violence by partner during pregnancy and use of prenatal care in rural India.	2011	Gender	2,877 rural married women aged 19–43 years from different demographic and socioeconomic contexts.	Bihar, Jharkhand, Maharashtra, Tamil Nadu	Quantitative data drawn from a prospective follow-up survey of original NFHS 2 respondents. Logistical regression models were fitted to the three binary outcomes of interest (receipt of prenatal care, receipt of a home-based prenatal check-up from a trained health worker, and receipt of at least three prenatal check-ups).	Women who experience physical violence during pregnancy are less likely to receive and measures of prenatal care, less likely to receive a home-based prenatal care check-up from a trained health worker, and less likely to receive three or more prenatal check-ups.
Begum et al. Association between domestic violence and unintended pregnancies in India.	2010	Gender	Married, pregnant women from the survey population (NFHS 2).		Data were drawn from the NHFS 2. Chi-square test was used to assess the association of each covariate, step-wise logistic regression analysis was done to fit the association between physical violence and unintended pregnancies.	After controlling for other variables women who were ever physically mistreated by their husbands were 47% more likely to experience unintended pregnancies.
Stephenson et al. Domestic violence, contraceptive use, and unwanted pregnancy in rural India.	2008	Gender	3,234 ever married women of reproductive age, with at least one child, and interviewed both for the NFHS 2 and the NFHS 2 follow up study conducted in 2002–2003.	Rural areas of Bihar, Jharkhand, Maharashtra, Tamil Nadu.	Two sets of data were used for this study: the NFHS 2 and the NFHS 2 follow-up survey conducted in 2002–2003. Data for both surveys were collected using structured questionnaires. Binary and logistic models were used in the analysis.	Women who reported experiencing physical domestic violence were significantly less likely to be using any contraceptive method and had significantly greater odds of experiencing an unwanted pregnancy than women not reporting domestic violence. The findings are consistent even after controlling for socioeconomic and demographic factors commonly found to influence the use of contraceptive behavior and pregnancy intentions.
Bahadur et al. Socio-demographic profile of women undergoing abortion in a tertiary center.	2008	Gender	118 women seeking abortion in the Family Planning Clinic at AIIMS in New Delhi.	New Delhi	Cross-sectional, retrospective, population-based study. Ways of analysis not described in the paper.	52.5% of the women had used some form of contraception prior to the current pregnancy. 34% had undergone an abortion in the preceding 2 years. Reasons for seeking abortions were unplanned pregnancy, inadequate income, contraception failure, and family being complete.
Ravindran and Balasubramanian. ‘Yes’ to abortion but ‘No’ to sexual rights: the paradoxical reality of married women in rural Tamil Nadu, India.	2004	Gender	66 ever married women and 44 of their husbands.	Rural Tamil Nadu	Data were collected using in-depth interviews. Ways of analysis was not described.	Low use of contraception and gender norms that prevent married women from refusing their husband's sexual demands lead to unplanned pregnancies and a subsequent need for abortion.
George. Persistence of high maternal mortality in Koppal District, Karnataka, India: observed services delivery constraints.	2007	Gender	The community of the Koppal District, Karnataka.	Koppal district, Karnataka	Data were collected through observations and semi-structured interviews with key stakeholders working with maternal health in the state of Karnataka and in the district of Koppal.	
Kulkarni and Chauhan. Socio-cultural aspects of reproductive morbidities among rural women in a district of Maharashtra, India.	2009	Gender	Six focus groups discussions (FGDs; 10–12 in each) with ever married women 18–45 years of age, living in both tribal and non-tribal areas, low SES	Nasik district, Maharashtra	Qualitative study using FGD.	Awareness on gynecological morbidities was low. Issues related to reproductive health (contraceptive use, health seeking) were considered a women's issue and not discussed with spouse, but husband is the one that makes decisions on treatment.
Chhabra. Sexual violence among pregnant women in India.	2008	Gender	2,000 pregnant women.		Interviews using semi-structured questionnaire.	30.7% of the women had been forced against their will to have sex with their partner during their last pregnancy.
Hall et al. Social and logistical barriers to the use of reversible contraception among women in a rural Indian village.	2008	Gender	75 married women 19+ years	Rural Maharashtra	Data were collected through FDGs and in-depth interviews. Analysis was conducted using MAXqda2 Software program.	Women felt that reversible contraceptives were undesirable, socially unacceptable, and unnecessary. Achievements of fertility goals are likely to be achieved due to female sterilization and with abortion as a back-up option.
Chacko. Women's use of contraception in rural India: a village-level study.	2001	Gender	600 married women randomly selected in the four villages constituting the study setting.	Rural West Bengal	A cross-sectional design was used to collect qualitative data. Bi- and multivariate analysis was conducted. Discriminant analysis was used to differentiate between users and non-users of modern contraceptives. Qualitative data were used to contextualize issues on use of contraceptives.	Patriarchal structures influence women's use of contraception; especially those women who married at a young age Power stratification in household can play a profound role in use of contraceptives.
Char et al. Influence of mother's-in-law on young couples’ family planning decision in rural India.	2010	Gender	60 households in which the couple and the mother in-law from each household were interviewed, in total 180 participants.	Rural Madhya Pradesh	Qualitative data were collected using open-ended questionnaires. Findings emerged using content analysis.	2/3 of the mother's in-law were of the opinion that they should take decisions on if and when to have an sterilization, and this was often related to when the number of son's required had been fulfilled. Both mother's in-law and couples said that mother's in-laws were left out of the discussion on the use of other type of contraceptives. Mother's in-law were concerned with side-effects of reversible methods, this perception was not shared by the couples.
Wilson-Williams. Domestic violence and contraceptive use in a rural Indian village.	2008	Gender	64 Hindu women with lower socioeconomic status and predominantly illiterate.	Rural Maharashtra	Data were collected using FGDs. Thematic analysis using MAXqda2 software was conducted.	Attitudes towards domestic violence are linked to women's ability to use contraception and to influence decisions in regards to family planning.
Bisoi et al. Correlations of anemia among pregnant women in a rural area of west Bengal.	2011	Education	219 pregnant women coming for ANC at subcenter.	Clinical setting, ANC clinic of Nasibpur, West Bengal.	Qualitative study collecting data on demographics and blood samples for hemoglobin levels.	67.8% of the women were anemic. High levels of anemia were shown to be associated with living in joint families, SES and level of education.
Singh et al. The use of contraceptives and unmet need for family planning in rural area of Patiala district.	2009	Education	1,123 ever married women, 15–49 years of age.	Rural area, District of Patiala, Punjab	Cross-sectional study. Data collected in clinical setting.	Most women (82.9%) belonged to a middle socio-economic class. 75.3% were using contraceptives at the time of the study. Education of wife influenced the contraceptive use.
Speizer et al. Family planning use among urban poor women from six cities of Uttar Pradesh, India.	2012	Education	17,643 currently married women, aged 15–49.	Six cities in Uttar Pradesh (Agra, Aligarh, Moradabad, Allahabad, Gorakhpur, Varanasi)	Both descriptive (univariate and bivariate) and multivariate regression is conducted.	Among women with low SES in both slum and non-slum areas: less educated women (1–11 years of education) are more likely to be sterilized, less likely to use modern contraceptives and more likely to have unmet demand for family planning than more educated women (+12 years of education).
Dwivedi and Sogarwal. Understanding contraceptive adoption in India: does women's autonomy matters?		Education	69,809 ever-married women, aged 15–49.	14 states in India	Sample drawn from the NFHS 2. Logistic regression was used to identify the net impact of physical, decision-making on the use of contraceptives.	The results show that higher levels of physical and economic autonomy indicates higher use of contraceptives. Association between contraceptive use and high levels of decision-making autonomy was not found. The results also show an increase in contraceptive use with literacy, but that the impact of level of education differs in different states.
Griffiths and Stephenson. Understanding users’ perspective of barriers to maternal health care use in Maharashtra, India.	2001	Education	45 women from both rural and urban settings with at least 2 children and the youngest being below the age of 5.	The cities of Pune and Mumbai, and the rural villages of Taleghar, Girawali, Mhalunge and Sikhwai	Data were generated though semi-structured interviews with open and closed questions. The respondents were recruited using a snowball sampling technique. Content analysis (Patton) was used to analyze the data.	Financial constraints are important when understanding the user's perspective of barriers to maternal health care but that these are also closely linked to perceptions of health care.
Kalyanwala et al. Abortion experiences of unmarried young women in India: Evidence from a facility-based study in Bihar and Jharkhand.	2010	Adolescence	549 unmarried women aged 15–24 who had an abortion in 2007–2008 at one of 16 clinics run by a NGO called Janani.	Bihar, Jharkhand (northern India)	Interviews using questionnaires was used to obtain data. Chi-square test and multivariate logistic regression analysis was used.	Women who lived in rural areas, those who did not receive full support from their partners and those who reported a forced encounter had an increased likelihood of having late abortions, while women who were older and who were better educated were more likely to have the abortion early in pregnancy.
Singh et al. Determinants of maternity care services utilization among married adolescents in rural India.	2012	Adolescence	3,599 women, ever married women in the age group 15–19, living in rural areas.	Rural India	Sample drawn from the NFHS 3. Bivariate analysis to determine the difference in proportion and logistic regression to understand the net effect of predictors variables on selected outcomes were applied.	Overall, 14% of the rural adolescent women received full and, 46% SKB, and 35% PNC. The rate of full ANC (7%) and PNC (24%) was low among uneducated women. Similarly, safe delivery care use was 31% among women with no formal education and 83% for those with high school education and above. The utilization of all three services was observed to increase with the increase in wealth quintile. Only 7% of the mother belonging to the poorest quintile received full ANC and 33% among the women from the richest wealth quintile.
Sahoo. Fertility behavior among adolescent in India.	2011	Adolescence	Ever married women, aged 15–19.	All India	Sample drawn from the DLHS 3. Bivariate and multivariate analysis was conducted.	High levels of child marriage. Low use of contraceptives and unmet demand for spacing methods.
Jejeebhoy et al. Experience seeking abortion among unmarried young women in Bihar and Jharkhand, India: delays and disadvantages.	2010	Adolescence	795 women seeking abortion were included in the survey, 26 of these women were randomly selected for in-depth interviews.	Bihar and Jharkhand, Clinical setting	Data were collected through a survey by using a questionnaire and through in-depth interviews. Logistic regression analysis was conducted.	80% of the respondents reported taking part of the decision of having an abortion, however, among the unmarried 14% and 4% reported that the decision was taken by parents and partner. A significant proportion of the women had made at least one unsuccessful attempt to terminate the pregnancy before coming to the clinic. Less than 1/3 of the unmarried and less than half of the married had confined in a family member or friend.
Raj et al. Prevalence of child marriage and its effect on fertility and fertility-control outcomes of young women in India: a cross-sectional, observational study.	2009	Adolescence	14,813 women, aged 20–24, ever married.		Data were drawn from the NFHS 3.Multiple regression was conducted.	Women married as children were significantly more likely to report no use of contraception before their first childbirth; more likely to have one or more unwanted pregnancies, pregnancy determination; and more likely to be sterilized than women married as adults.
Prakash et al. Early marriage, poor reproductive health status of mother and child wellbeing in India.	2011	Adolescence	39,026 married women, aged 15–49, gave birth up to 5 years prior to the data collection for the NFHS 3.		Data were drawn from the NFHS 3. Bivariate analysis, multiple linear regressions was conducted.	The results from the multiple regression show that women married as children are more likely to have poorer reproductive health than women married as adults.
Singh, Rai and Singh. Assessing the utilization of maternal and child health care among married adolescent women: evidence from India.	2012	Adolescence	Sample of 5,253 married women that had experience pregnancy during adolescence drawn from the NFHS 3.	29 States	Data were drawn from the NFHS 3. Bivariate and multivariate analysis was conducted.	10% of the adolescent women utilized ANC, around 50% used safe delivery services.
Rao S, Joshi et al. Social dimensions related to anemia among women of childbearing age from rural India.	2010	Adolescence	418 non-pregnant women aged 15–35, most of the women living in farming communities	Three villages (Dhamari, Hivare and Pimple) in the Pune District in the state of Maharashtra	Cross-sectional study. A structure questionnaire was used to obtain personal information, obstetric history, dietary assessment and personal information. Data on weight, height and blood sample obtained. Multiple logistic regression model analysis was carried.	77% of the women were suffering from some degree of anemia. The risk of iron-deficiency anemia was higher among women with lower body weight and short maternal height, younger age at marriage and higher parity. Various socio-culture reasons associated with low consumption of green leafy vegetables were found.
Mukhopadhyay et al. Hospital-based perinatal outcomes and complications in teenage pregnancy in India.	2010	Adolescence	350 adult and 350 adolescent mothers	RG Kar Medical College and Hospital in Kolkata	Cross-sectional study. Data were collected through interviews and by observations using pretested schedule.	Adolescent mothers were shown to have a higher proportion (27%) of preterm deliveries compared to 13.1% in the adult mothers. Adolescent mothers developed more adverse prenatal complications such as preterm birth, stillbirths, neonatal deaths, and delivered low birth weight babies when compared adult primigravida mothers.
Santhya et al. Associations between early marriage and young women's marital and reproductive health outcomes: evidence from India.	2010	Adolescence	8,314 married women, aged 20–24 at the time of interview	Rural and urban areas of Andhra Pradesh, Bihar, Jharkhand, Maharashtra, Rajasthan and Tamil Nadu.	Sample for this study is drawn from a survey conducted in the four states during 2006–2008.	Young women who had married at age 18 or older were more likely than those who had married before the age of 18 to have been 1) involved in planning their marriage (odds ratio 1,4), 2) to reject wife beating (OD 1,2), 3) to have used contraceptives to delay first pregnancy (OD 1.4), and 4) to have had their first delivery in an institution.
Trivedi and Pasrija. Teenage pregnancies and their obstetric outcomes.	2007	Adolescence	840 women aged 13–19 attending ANC or admitted to the maternity ward, mostly belonging to a poor households	Urban New Delhi	Data were collected in a hospital in New Delhi. A control group of women above the age of 20 attending ANC or/and were admitted to the maternity ward was used for comparison. Descriptive statistics were used.	Adolescents in the study were found to be at higher risk for severe anemia, eclampsia and preterm labor compared to non-adolescent women.

## References

[1] Registrar General, India (2011). Special bulletin on maternal mortality in India 2007–09. Sample registration system bulletin.

[2] Östlin P, Schrecker T, Sadana R, Bonnefoy J, Gilson L, Hertzman C (2011). Priorities for research on equity and health: towards an equity-focused health research agenda. PLoS Med.

[3] Say L, Raine R (2007). A systematic review of inequalities in the use of maternal health care in developing countries: examining the scale of the problem and the importance of context. Bull World Health Organ.

[4] Culyer AJ (2001). Equity – some theory and its policy implications. J Med Ethics.

[5] Withhead M, Dahlgren G (2006). Levelling up (part1): a discussion on concepts and principles for tackling social inequities in health. Studies on Social and Economic Determinants of Population Health No. 2. Report number WHOLIS E89383.

[6] Solar O, Irwin A (2010). A conceptual framework for action on the social determinants of health. Social Determinants of Health Discussion Paper 2 (Policy and Practice). Report number WA 525.

[7] Tendulkar SD, Radhakrishna R, Sengupta S (2009). Report of the expert group to review the methodology for estimation of poverty. Government of India, Planning Commission.

[8] Berman P, Ahuia R, Bhandari L (2010). The impoverishing effect of health care payments in India: new methodology and findings. Econ Pol Weekly.

[9] Balarajan Y, Selvaraj S, Subramanian SV (2011). Health care and equity in India. Lancet.

[10] World Bank (2011). Main report vol 2. Social protection for a changing India. Report No. 61275. http://www-wds.worldbank.org/external/default/WDSContentServer/WDSP/IB/2011/04/20/000333037_20110420235739/Rendered/PDF/612750v20ESW0P11SP0Report0Volume0II.pdf.

[11] Pathak PK, Singh A, Subramanian SV (2010). Economic inequalities in maternal health care: prenatal care and skilled birth attendance in India, 1992–2006. PLoS One.

[12] Mohanty SK, Pathak PK (2009). Rich-poor gap in utilization of reproductive and child health services in India, 1992–2005. J Biosoc Sci.

[13] Singh A, Becker S (2012). Concordance between partners in desired waiting time to birth for newlyweds in India. J Biosoc Sci.

[14] Gupta M, Thakur JS, Kumar R (2008). Reproductive and child health inequalities in Chandigarh Union Territory of India. J Urban Health.

[15] Bonu S, Bhushan I, Rani M, Anderson I (2009). Incidence and correlates of ‘catastrophic’ maternal health care expenditure in India. Health Policy Plan.

[16] Dhar R, Nagpal SJ, Sinha S, Bhargava VL, Sachdeva A, Bhartia A (2009). Direct cost of maternity-care services in South Delhi: a community survey. J Health Popul Nutr.

[17] Rani M, Bonu S, Harvey S (2008). Differentials in the quality of antenatal care in India. Int J Qual Health Care.

[18] Dhar RS, Nagpal J, Bhargava V, Sachdeva A, Bhartia A (2010). Quality of care, maternal attitude and common physician practices across the socio-economic spectrum: a community survey. Arch Gynecol Obstet.

[19] Griffiths P, Stephenson R (2001). Understanding users’ perspectives of barriers to maternal health care use in Maharashtra, India. J Biosoc Sci.

[20] Hazarika I (2010). Women's reproductive health in slum populations in India: evidence from NFHS-3. J Urban Health.

[21] Kumar M, Meena J, Sharma S, Poddar A, Dhalliwal V, Modi M-S C (2011). Contraceptive use among low-income urban married women in India. J Sex Med.

[22] Kumar A, Mohanty SK (2011). Intra-urban differentials in the utilization of reproductive healthcare in India, 1992–2006. J Urban Health.

[23] Skordis Worrall J, Pace N, Bapat U, Das S, More NS, Joshi W (2011). Maternal and neonatal health expenditure in Mumbai slums (India): a cross sectional study. BMC Public Health.

[24] Das S, Bapat U, More NS, Chordhekar L, Joshi W, Osrin D (2010). Prospective study of determinants and costs of home births in Mumbai slums. BMC Pregnancy Childbirth.

[25] Agrawal S, Sethi V, Srivastava K, Jha PK, Baqui AH (2010). Birth preparedness and complication readiness among slum women in Indore city, India. J Health Popul Nutr.

[26] Bhanderi MN, Kannan S (2010). Untreated reproductive morbidities among ever married women of slums of Rajkot City, Gujarat: the role of class, distance, provider attitudes, and perceived quality of care. J Urban Health.

[27] International Institute for Population Sciences and Macro International (2007). National Family Health Survey (NFHS-3), 2005–2006. India.

[28] Kesterton AJ, Cleland J, Sloggett A, Ronsmans C (2010). Institutional delivery in rural India: the relative importance of accessibility and economic status. BMC Pregnancy Childbirth.

[29] Gupta SD, Khanna A, Gupta R, Sharma NK, Sharma ND (2010). Maternal mortality ratio and predictors of maternal deaths in selected desert districts in rajasthan a community-based survey and case control study. Womens Health Issues.

[30] Varkey P, Balakrishna PP, Prasad JH, Abraham S, Joseph A (2000). The reality of unsafe abortion in a rural community in South India. Reprod Health Matters.

[31] Iyengar K, Iyengar SD, Suhalka V, Dashora K (2009). Pregnancy-related deaths in rural Rajasthan, India: exploring causes, context, and care-seeking through verbal autopsy. J Health Popul Nutr.

[32] Chaturvedi S, Ranadive B (2007). Are we really making motherhood safe? A study of provision of iron supplements and emergency obstetric care in rural Maharashtra. Natl Med J.

[33] Noronha JA, Bhaduri A, Vinod Bhat H, Kamath A (2010). Maternal risk factors and anaemia in pregnancy: a prospective retrospective cohort study. J Obstet Gynaecol.

[34] Patra S, Puri M, Trivedi SS, Pasrija S (2010). Clinical profile of women with severe anemia in the third trimester of pregnancy. Trop Doct.

[35] Hausmann R, Tyson LD, Zahidi S (2011). The global gender gap report 2011.

[36] International Institute for Population Sciences and Macro International (2009). Gender equality and women's empowerment in India. National Family Health Survey (NFHS-3), 2005–06. India.

[37] Mistry R, Galal O, Lu M (2009). Women's autonomy and pregnancy care in rural India: a contextual analysis. Soc Sci Med.

[38] Saika N, Singh A (2009). Does type of household affect maternal health? Evidence from India. J Biosoc Sci.

[39] Allendorf K (2010). The quality of family relationships and use of maternal health-care services in India. Stud Fam Plann.

[40] Char A, Saavala M, Kulmala T (2010). Influence of mothers-in-law on young couples’ family planning decision in rural India. Reprod Health Matters.

[41] Koski AD, Stephenon R, Koenig MR (2011). Physical violence by partner during pregnancy and use of prenatal care in rural India. J Health Popul Nutr.

[42] Chhabra S (2008). Sexual violence among pregnant women in India. J Obstet Gynaecol Res.

[43] Begum S, Dwivedi SN, Pandey A, Mittal S (2010). Association between domestic violence and unintended pregnancies in India: findings from the National Family Health Survey-2 data. Natl Med J India.

[44] Stephenson R, Koenig MA, Acharya R, Roy TK (2008). Domestic violence, contraceptive use, and unwanted pregnancy in rural India. Stud Fam Plann.

[45] Wilson-Williams L, Stephenson R, Juvekar S, Andes K (2008). Domestic violence and contraceptive use in a rural Indian village. Violence Against Women.

[46] Hall MA, Stephenson RB, Juvekar S (2008). Social and logistical barriers to the use of reversible contraception among women in a rural Indian village. J Health Popul Nutr.

[47] Chacko E (2001). Women's use of contraception in rural India: a village-level study. Health Place.

[48] Kulkarni K, Chauhan S (2009). Socio-cultural aspects of reproductive morbidities among rural women in a district of Maharashtra, India. J Fam Welf.

[49] Bahadur A, Mittal S, Sharma JB, Sehgal R (2008). Socio-demographic profile of women undergoing abortion in a tertiary center. Arch Gynecol Obstet.

[50] Ravindran TK, Balasubramanian P (2004). “Yes” to abortion but “no” to sexual rights: the paradoxical reality of married women in rural Tamil Nadu, India. Reprod Health Matters.

[51] George A (2007). Persistence of high maternal mortality in Koppal district, Karnataka, India: observed service delivery constraints. Reprod Health Matters.

[52] Chandramouli C (2011). Census of India 2011. Provisional population totals, paper 1 of 2011, India, Series 1.

[53] Jat TJ, Ng N, Sebastian MS (2011). Factors affecting the use of maternal health services in Madhya Pradesh state of India: a multilevel analysis. Int J Equity Health.

[54] Speizer IS, Nanda P, Achyut P, Pillai G, Guilkey DK (2012). Family planning use among urban poor women from six cities of uttar pradesh, India. J Urban Health.

[55] Dwivedi LK, Sogarwal R (2008). Understanding contraceptive adoption in India: does women's autonomy matters?. J Fam Wel.

[56] Singh N, Kaur G, Singh J (2009). The use of contraceptives and unmet need for family planning in rural area of Patiala district. J Fam Welf.

[57] Bisoi S, Haldar D, Majumdar TK, Bhattacharya N, Sarkar GN, Ray SK (2011). Correlations of anemia among pregnant women in a rural area of West Bengal. J Fam Welf.

[58] Thind A, Mohani A, Banerjee K, Hagigi F (2008). Where to deliver? Analysis of choice of delivery location from a national survey in India. BMC Public Health.

[59] Institute for Population Science and Population Council (2010). Youth in India: situation and needs 2006–2007.

[60] Jejeebhoy SJ, Kalyanwala S, Zavier AJ, Kumar R, Jha N (2010). Experience seeking abortion among unmarried young women in Bihar and Jharkhand, India: delays and disadvantages. Reprod Health Matters.

[61] Kalyanwala S, Zavier AJ, Jejeebhoy S, Kumar R (2010). Abortion experiences of unmarried young women in India: evidence from a facility-based study in Bihar and Jharkhand. Int Perspect Sex Reprod Health.

[62] Sahoo H (2011). Fertility behaviour among adolescent in India. J Fam Welf.

[63] Prakash R, Singh A, Pathak PK, Parasuraman S (2011). Early marriage, poor reproductive health status of mother and child well-being in India. J Fam Plann Reprod Health Care.

[64] Raj A, Saggurti N, Balaiah D, Silverman JG (2009). Prevalence of child marriage and its effect on fertility and fertility-control outcomes of young women in India: a cross-sectional, observational study. Lancet.

[65] Trivedi SS, Pasrija S (2007). Teenage pregnancy and their obstetric outcomes. Trop Doct.

[66] Singh PK, Rai RK, Alagarajan M, Singh L (2012). Determinants of maternity care services utilization among married adolescents in rural India. PLoS One.

[67] Santhya KG, Ram U, Acharya R, Jejeebhoy SJ, Ram F, Singh A (2010). Associations between early marriage and young women's marital and reproductive health outcomes: evidence from India. Int Perspect Sex Reprod Health.

[68] Singh L, Rai KR, Singh Pk (2012). Assessing the utilization of maternal and child health care among married adolescent women: evidence from India. J Biosoc Sci.

[69] Mukhopadhyay P, Chaudhuri RN, Paul B (2010). Hospital-based perinatal outcomes and complications in teenage pregnancy in India. J Health Popul Nutr.

[70] Rao S, Joshi S, Bhide P, Puranik B, Kanade A (2011). Social dimensions related to anaemia among women of childbearing age from rural India. Public Health Nutr.

[71] Mukherjee S, Haddad S, Narayana D (2011). Social class related inequalities in household health expenditure and economic burden: evidence from Kerala, South India. Int J Equity Health.

[72] Nayar KR (2007). Social exclusion, caste and health: a review based on the social determinants of health framework. Indian J Med Res.

[73] Maiti S, Unisa S, Agrawal PK (2005). Health care and health among tribal women in Jharkhand: a situational ananlysis. Stud Tribes Tribals.

[74] Saroha E, Altarac M, Sibley LM (2008). Caste and maternal health care service use among rural Hindu women in Maitha, Uttar Pradesh, India. J Midwifery Womens Health.

[75] Hazarika I (2011). Factors that determine the use of skilled care during delivery in India: implications for achievement of MDG-5 targets. Matern Child Health J.

[76] Agrawal PK, Agrawal S (2010). To what extent are the indigenous women of Jharkhand, India living in disadvantageous conditions: findings India's National Family Health Survey. Asian Ethnicity.

[77] Basu S, Kapoor AK, Basu SK (2004). Knowledge, attitude and practice of family planning among tribals. J Fam Welf.

[78] Lakshmi G, Sambasiva Rao R, Giridhar L (2011). Perceptions towards family planning – a study on tribal women from Andhra Pradesh. Indian J Matern Child Health.

[79] Bhasin MK, Nag S (2007). Demography of the tribal groups of Rajasthan: 5. Dynamics of family planning methods usage. Anthropologist.

[80] Deb R (2010). Knowledge, attitude and practices related to family planning methods among the Khasi Tribes of East Khasi hills Meghalaya. Anthropologist.

[81] Tehreek-E Pasmanda Muslim Samaj (2008). Millennium development goals and Muslims – a status report.

[82] Tuddenham SA, Rahman MH, Singh S, Barman D, Kanjilal B (2010). Care seeking for postpartum morbidities in Murshidabad, rural India. Int J Gynaecol Obstet.

[83] Jeffery P, Jeffery R (2010). Only when the boat has started sinking: a maternal death in rural North India. Soc Sci Med.

[84] Raj A, Sabarwal S, Decker MR, Nair S, Jethva M, Krishnan S (2011). Abuse from in-laws during pregnancy and post-partum: qualitative and quantitative findings from low-income mothers of infants in Mumbai, India. Matern Child Health J.

[85] Diderichsen F, Evans T, Whitehead M, Evans T (2001). The social basis of disparities in health. Challenging inequities in health: from ethics to action.

[86] Jejeebhoy S, Sathar Z (2001). Women's authonomy in India and Pakistan: the influence of religion and region. Pop Dev Review.

[87] Sen A (2001). Gender equity and the population problem. Int J Health Serv.

[88] Sen G, Iyer A (2012). Who gains, who loses and how: leveraging gender and class intersections to secure health entitlements. Soc Sci Med.

[89] Iyer A, Sen G, George A (2007). The dynamics of gender and class in access to health care: evidence from rural Karnataka, India. Int J Health Serv.

[90] Nyamtema AS, Urassa DP, van Roosmalen J (2011). Maternal health interventions in resource limited countries: a systematic review of packages, impact and factors for change. BMC Pregnancy and Childbirth.

[91] Lim SS, Dandona L, Hoisington JA, James SL, Hogan MC, Gakidou E (2010). India's Jani Suaksha Yojana, a conditional cash transfer programme to increase birth in health facilities: an impact evaluation. Lancet.

[92] United Nations Children's Fund (2012). State of world's children 2012 – Children in an urban world.

